# Community integration, quality of life, thriving, and mental health among refugees and asylum seekers. A London service provider perspective

**DOI:** 10.3389/fpubh.2024.1358250

**Published:** 2024-04-18

**Authors:** Hanna Kienzler

**Affiliations:** Department of Global Health & Social Medicine, Faculty of Social Science & Public Policy, King’s College London, London, United Kingdom

**Keywords:** refugees, community integration, quality of life, thriving, mental health, racism, asylum system, asylum seekers

## Abstract

**Introduction:**

This article explores how systemic injustices and social inequalities affect refugee and asylum seeker integration, thriving, and mental health in London. This is pertinent as the United Kingdom currently operates a ‘broken’ asylum system with unfair policies and a ‘tough’ immigration rhetoric which makes it extraordinarily difficult for asylum seekers and refugees to achieve community integration, have a good quality of life, be able to thrive, and have good health including mental health. Paradoxically, the United Kingdom Home Office also features an Indicators for Integration Framework to provide practical ways to design more effective strategies, monitor services and evaluated integration interventions.

**Methods:**

This study employed a qualitative research design including semi-structured interviews with 19 mental health and psychosocial support service providers working in third-sector organizations in London.

**Results:**

The study results show that the current asylum system severely undermines efforts to support asylum seekers and refugees with their integration. All participants highlighted that asylum seekers and refugees lacked experienced poor quality of life and faced structural challenges to build meaningful social connections; to have access education, fair employment and good work; to achieve good mental health and wellbeing; and to be able to thrive.

**Discussion:**

To improve community integration, quality of life, thriving, and mental health for asylum seekers and refugees in London and, beyond, the United Kingdom, four recommendations are made on structural and service-levels: (1) reform of the current asylum system by centering human rights; (2) implement and carry out needs assessments among asylum seekers and refugees focussing on key social determinants; (3) ensure asylum seekers and refugees benefit from the NHS Inclusion Health framework; and (4) extend the NHS Patient and Carer Race Equality framework beyond England. To be effective, all four initiatives need to be grounded in a participatory approach that meaningfully involves diverse groups of stakeholders including asylum seekers and refugees.

## 1 Introduction

At the end of June 2023, 110 million people world-wide were forcibly displaced as a result of persecution, conflict, violence, human rights violations, and events seriously disturbing public order (1). The latest UNHCR Mid-Year Trends report states that more than 1 in 73 people are forcibly displaced with the majority (approximately 9 in 10) living in low-and middle-income countries. Countries where most of the world’s refugees come from include Syria, Afghanistan, Ukraine, Venezuela, and South Sudan while the top receiving countries are Iran and Türkiye, followed by Germany, Colombia, and Pakistan. In the UK, in contrast, people seeking asylum make up a small proportion of new arrivals with approximately 75,340 applications made in the year ending September 2023 (2). It is estimate that London supported around 8,455 asylum seekers in 2021, which is the fewest relative to its population, with just over 1 person per 10,000, compared to other regions in the United Kingdom (3, 4). The Refugee Council indicates that the United Kingdom ranks 20th highest in Europe in terms of the number of asylum applications per head of population. Among those claiming asylum, three-quarters are granted protection at the initial stage and refusals are often overturned on appeal (2).

The United Kingdom has been accused of operating a “broken” asylum system with unfair policies and a “tough” immigration rhetoric which makes it extraordinarily difficult for asylum seekers^1^ and refugees to achieve community integration, have a good quality of life, be able to thrive, and have good health including mental health (5, 6). In 2012, the country implemented a “hostile environment” policy, spearheaded by the then Conservative Home Secretary Theresa May. The policy refers to several measures aimed at “identifying and reducing the number of immigrants in the United Kingdom with no right to remain” (7). Measures initially included to restrict “illegal immigrants” renting property, driving, having bank accounts, and accessing benefits and free healthcare. Some of these initiatives included data-sharing between other government departments or external organizations and the Home Office, and the requirement for document checks by certain service providers. The “hostile environment” has been extended by the Nationalities and Borders Act 2022, the Illegal Migration Act 2023, and the much-debated Rwanda Plan. These reforms introduced a two-tier asylum process that distinguishes people based on how they arrive to the United Kingdom (8). They give more negative consideration to those who arrive by irregular routes such as via small boats, on the backs of lorries, or overstaying their visas. Those deemed inadmissible to the UK’s asylum system are now either returned to their home countries or potentially eligible for deportation to Rwanda (and to other ‘safe’ third countries) where they must seek asylum instead (9). Those allowed to seek asylum in the United Kingdom frequently wait years for a decision on their leave to remain. The backlog at the end of June 2023 was nearly 173,000 people awaiting an initial decision on their asylum claim with almost three quarters of people waiting for more than 6 months (10).

**Figure 1:**
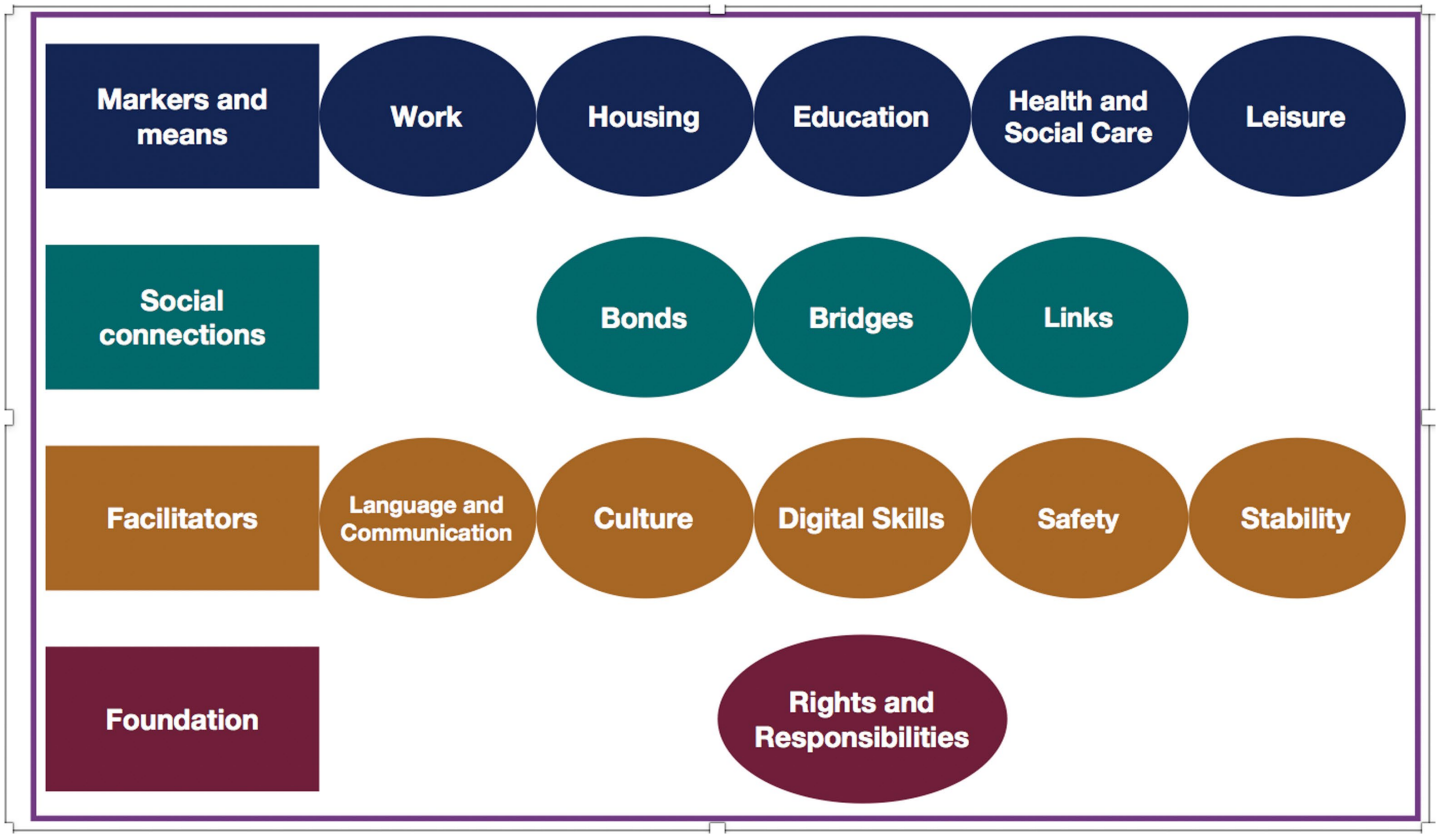
Home Office Indicators of Integration Framework (11).

Paradoxically to the “hostile environment” policy, the United Kingdom’s Home Office has also designed and published an Indicators of Integration Framework “to inform the planning, monitoring and evaluation of integration projects” ((11), p.7). Its foreword explicitly states that “successful integration helps people to realize their full potential. It makes it easier for them to access services, reduces educational and health inequalities, helps them to find jobs and, fundamentally, underpins social cohesion and community empowerment” (p. 7). Its vision for integration is “communities where people, whatever their background, live, work, learn and socialize together, based on shared rights, responsibilities, and opportunities” (p. 11). As such integration of refugees and migrants is considered to be (a) multi-dimensional as it depends on multiple factors encompassing access to resources and opportunities as well as social mixing; (b) multi-directional in that it involves adjustments by everyone in society; (c) depending on everyone taking responsibility for their own contribution including newcomers, receiving communities, and government; and (e) context specific as it needs to be understood and planned in relation to its particular context and within a bespoke timeframe (P.11). The Indicators of Integration framework is structured around 14 key domains grouped according to (1) markers and means (work, housing, education, health and social care, leisure); (2) social connections (bonds with people sharing similar backgrounds and experiences, bridges into the host community, links to services and support organizations); (3) facilitators (language and communication, culture, digital skills, safety, stability); and (4) foundation (rights and responsibilities) (see Figure 1). Progress in these domains depends on the contribution of members of receiving communities and local institutions as well as the asylum seekers, refugees, or other migrants. It is considered to lead to greater community integration, better quality of life, and improved health, including mental health.

This article explores how the 14 domains of the United Kingdom Indicator of Integration Framework are experienced by asylum seekers and refugees and the ways in which these experiences affect their quality of life, thriving, and mental health, from the perspective of mental health and psychosocial support service providers working in London. The findings will be interpreted through a conceptual framework that brings together social determinants of health, quality of life, structural violence, and systems of oppression. Social determinants of health are the non-medical factors that determine “the conditions in which people are born, grow, work, live, and age, and the wider set of forces and systems shaping the conditions of daily life” (12). Social determinants have an important influence on health by affecting people’s quality of life which the World Health Organization (WHO) defines as “an individual’s perception of their position in life in the context of the culture and value systems in which they live and in relation to their goals, expectations, standards and concerns” (13). This wide-ranging concept brings together people’s physical and psychological state, independence, social relationships, beliefs, and their relationships to salient environmental features which, if aligned, enable healthy, comfortable, enjoyable lives. For people from marginalized communities, these states are often prevented from aligning due to entrenched social structures characterized by poverty and steep grades of social inequalities. The underlying drivers have been considered a form of structural violence committed by “the social machinery of oppression” which bolsters oppressive systems such as racism, xenophobia, sexism, heterosexism, ableism, classism, and ageism ((14), p.307). Those most affected tend to be marginalized groups (e.g., racial and minoritised ethnic groups, asylum seekers and refugees, LGTQI+ people) as imbalances in power, wealth, and opportunity are sustained and, in turn, fuel “profoundly disparate health outcomes within and between communities” (15). In fact, long-standing intersectional systems of oppression have been shown to put individuals at greater risk for contracting certain conditions as they can disrupt physiological processes essential to maintaining good health while causing great distress which impacts on people’s mental health.

Systems of oppression and the structural violence they cause form an integral part of the United Kingdom asylum system. Research has shown that complex and prolonged legal procedures to claim asylum and overall immigration policies negatively affect the social determinants and quality of life of those awaiting asylum processing (16, 17). Currently, around 37,000 asylum seekers are accommodated in sub-standard housing such as squalid house shares, dilapidated hotels and hostels, disused military barracks, and, most recently, the controversial Bibby Stockholm barge (Human Rights (18); see also (19)). Asylum seekers are not permitted to work before 12 months into their application process and have no access to public funds for ESOL (English for Speakers of other Language) classes for the first 6 months (20). Without employment, asylum seekers are at risk of becoming homeless and destitute as any savings they have must be spent on basic necessities. Furthermore, asylum seekers face restrictions related to claiming mainstream welfare benefits, renting private accommodation, education and vocational training, opening bank accounts, or accessing free secondary healthcare (21). Those who have gained asylum have only 28 days until their asylum support expires, during which time they must find employment and private accommodation or apply for welfare and housing support (Citizens (22, 23)). In London, this transition period has been shown to detrimentally affect community inclusion due to limited integration support and the heightened risk of homelessness as new refugees tend to lack savings while facing high up-front costs of tenancy deposits. The Refugee Council states: “When someone in London is granted asylum, they face a crisis situation as the lack of integration support for new refugees intersects with the London housing crisis. This is particularly acute for those new refugees seeking to find a private tenancy” ((3), p. 3).

While widely recognized that the quality of life of asylum seekers and refugees is severely compromised, less information is available about how this affects mental health outcomes among those with lived experience (24). Nevertheless, the United Kingdom government does recognize an increased risk of mental health problems among asylum seekers and refugees. Data for England shows that they are five times more likely to have mental health needs than the general population and over 61% will experience serious mental distress (25, 26). Studies outside the United Kingdom report pre-migration experiences of adversity and trauma are linked to high prevalence of PTSD (31%) and depression (11%) among asylum seekers and refugees persisting for years after immigration (23). Post-migration studies show associations with substance-use disorders and psychosis. However, asylum seekers and refugees are not equally affected by mental health problems in that women and girls, people with disabilities, those experiencing discrimination and racism, and who have lower socioeconomic status carry the greater burden (23).

In the UK, mental distress is further amplified as accessing mental health care and psychosocial support are challenging. In England, although primary care is free, refused asylum seekers or undocumented migrants pay for secondary care unless deemed ‘urgent’ or ‘immediately necessary’ (21). This is also the case for NHS community health services except for those detained under the Mental Health Act (17). Asylum seekers and refugees report discrimination, intersecting with race and disability, when seeking mental health care in the NHS. Language issues and interpretation services, inconsistency of care, lack of trust in mental health providers, and concerns about charging also affect access (27). A parallel network of charity organizations exists that seek to mitigate these shortcomings offering mental health and psychosocial support and help with accessing more specialized services including those provided by the NHS (21). At the same time, research shows that mental health services needs could be reduced by providing asylum seekers and refugees with better social support and opportunities for community integration (17). However, important insight is lacking into the intersections between policies promoting community integration, quality of life, and mental health outcomes among asylum seekers and refugees and what it would take to ensure people can settle and even thrive in host communities.

## 2 Methods

This study employed a qualitative research design to explore London-based service providers’ beliefs, experiences, and views with regards to how community inclusion, social support, and thriving are linked to asylum seeker and refugee mental health. The study was carried out in summer 2020.

Study participants included 19 mental health and psychosocial support service providers who were predominantly female except for four male participants. They all worked with non-governmental organizations in different roles including director or CEO (6); manager (operations, community integration, clinical, therapeutic service) (5); advisor (policy and practice, welfare benefits, housing, employment) (2); psychotherapist (2); mental health counselor (2); arts therapist (1); and mental health advocate (1). The organizations offered a combination of services including social support (e.g., social activities, training courses, career guidance, mentoring for children and adolescents, language training, housing); legal advice (e.g., immigration, help with settlement or citizens applications); family support (e.g., activities for parents and children, after-school activities, counseling on marriage, family dispute and domestic violence); and wellbeing-focussed services (e.g., community support groups, emotional support, mental and physical health support, help accessing healthcare services). Some services were provided to all groups, others supported people from a particular nationality or ethnic group, or were specifically designed for women, children, adolescents, or older people.

Sampling for this study followed a purposeful and snowball sampling approach. Invitation emails were sent to 129 non-governmental organizations providing mental health and psychosocial support services to asylum seekers and refugees in London (later collated in a directory^2^). Reponses were followed up with information sheets and consent forms. Moreover, at the end of each interview, participants were asked to provide contact information to other service providers working in the similar fields. However, all suggested service providers were working for organizations that had already been contacted. While most organizations did not respond to our request, those who did also decided to participate in interviews, with the exception of three organizations who indicated that they lacked capacity to do so. The relatively low response rate might have been due to the Covid-19 pandemic as organizations were busy adapting their way of working in line with government-imposed restrictions while trying to meet the needs of their often very vulnerable beneficiaries.

Semi-structured interviews were carried out following a topic guide about service provision, mental health profile of beneficiaries, community, integration, social exclusion, and thriving. Example interview questions around which conversations ensued included, but were not limited to: (a) Community inclusion: What do you think does community mean to asylum seekers and refugees? How do you think asylum seekers and refugees build a sense of community in the UK? What might some of the challenges be? (b) Social exclusion: According to your opinion, how do asylum seekers and refugees experience social exclusion? Based on your insights, how do they cope with social exclusion? (c) Support: What do you think does support mean to asylum seekers and refugees? According to your insights, what do their social support networks consist of? What kind of support are they most likely to receive? What are the barriers to getting support for asylum seekers and refugees? How do they overcome these barriers? (d) Thriving: According to your understanding, what does thriving mean to asylum seekers and refugees? What enables them to thrive? What else would be needed to help asylum seekers and refugees to thrive? Each topic included several probes to explore mental health impacts and particular mental health related challenges.

The interviews were conducted online due to pandemic restrictions, lasted approximately 1 hour and were, with the consent of participants, audio recorded and later transcribed verbatim. The data from the transcripts were subsequently analysed using thematic analysis following the approach proposed by Clark and Braun (28) consisting of familiarization with the data and a combination of inductive and deductive coding to capture the semantic and conceptual reading of the data, and to build categories and themes from the codes. Deductive codes were informed by the domains of the Indicators of Integration Framework. In this process, the coded data were, first, categorized and linked by relationship. In a next step, links were established between the categories so that overarching themes could be identified that structure the following results section.

Ethics approval for this study was obtained from King’s College London Research Ethics Committee (reference number: MRA-19/20–20750).

## 3 Results

The results will focus on the three key areas derived from thematic analysis including: (a) the meanings of community and community integration; (b) the domains of the Indicators of Integration Framework and how they are experienced by asylum seekers and refugees with particular attention to their effects on quality of life and mental health; and (c) experiences of thriving in the hostile environment. Within each of these themes, service providers discuss effects on mental health and highlight recommendations of what could enable greater integration, quality of life, thriving, and improved mental health for refugees and asylum seekers in the United Kingdom.

### 3.1 The meanings of community and community integration

To understand what community integration means, our interviews began by unpacking the concept of “community.” Service providers considered “community” for refugees and asylum seekers to be something dynamic, multi-faceted, and context dependent. They associated it with geographic place, social connection, belonging, familiarity, identity, support, and emotions. A clinical manager said: “It’s something about belonging, familiarity, having a sense of place, feeling responsible and responsive to your environment and the people around you.” As such, community was perceived to be something positive in that it brought people together in supportive structures.

At the same time, however, it was considered difficult to access due to exclusionary attitudes of people in host communities including racism and other forms of discrimination. It was mentioned that “the community might not be very welcoming and friendly anyway” (Director of an organization). Further barriers to becoming part of a community were linked to people’s legal status and, connected to this, their ability to “put down roots” and “connect with others.” For example, asylum seekers were often moved from one part of the country, city, or neighborhood to another while not knowing whether their asylum application would be successful. A clinical manager said:

When you are an asylum seeker, it is particularly tricky (…) because the anxiety of not knowing whether you’re going to be able to stay in the UK. [This] is also a deterrent about getting too attached to others. So, there’s this sort of longing to have more connections and more support, but at the same time, there’s a sort of fear about getting stuck in case (…) you get deported.

These worries were linked to feelings of isolation and mental health problems such as depression and anxiety. Mental health problems, in turn, were described as a further barrier to integration into community as people lacked motivation to reach out to others and isolated themselves even further out of fear of stigma from fellow refugees and the wider community, fear to register with health and social services worrying they might get deported, and language and cultural barriers when accessing services.

### 3.2 Experiences of key domains of the indicators of integration framework

#### 3.2.1 Foundations for community integration

When asked what it took for refugees and asylum seekers to be included in the community, all service providers highlighted the importance of a well-functioning asylum system and rights. The asylum system was considered to be “broken,” “unfair,” “inefficient,” and deeply “dehumanizing.” There was a tacit agreement that the situation had worsened over the past years affecting the asylum sector, as a whole, and asylum seekers, in particular, detrimentally. A participant said: “You know, let us get rid of all these hostile environment policies that have been building just layer upon layer over the last 10 years and before and just giving people basic rights and entitlements that a system should have.” Particularly detrimental were the long waiting times that asylum seekers had to endure before their asylum applications were processed. These were linked to the development or worsening of mental health problems such as PTSD, depression, and anxiety as well as hopelessness that could culminate in suicidal ideation and even suicide. An operations manager said: “This is why that increase, they are all really have got, they have got PTSD, they have got anxiety and, for example, asylum seekers, while they are waiting to get a decision, you know, the length of waiting to get decision, that is one part that creates lots of anxiety, depression and lots of issues, you know, because they do not know, they can be deported anytime, you know.”

While in the asylum application process, asylum seekers were seen as living “in limbo” as they had “lost their roots” back home while being unable to “fully arrive” in the host community. They were described to be in a liminal space, neither quite here nor there. The feeling of being in limbo was directly connected to the uncertainties emanating from the hostile environment policies and, related to this, the lack of rights and entitlements to work and to access education, adequate housing, general welfare, and specialized healthcare. The director of an organization said: “So, it’s exclusion from entitlement like welfare and benefits, its exclusion from being able to choose what housing you live in. It’s exclusion from education (…), not having the right to work and not being able to travel. (….) [It’s] financial exclusion – having such limited asylum seeker support means you can literally just survive and do very little else.” Living in limbo was, thus, connected to feelings of uncertainty, insecurity, hopelessness, and wasting one’s best years. A mental health counselor said, trying to put themselves into the shoes of asylum seekers: “(…) if you lived 13 years of your life in this country and you are not able to go and study, they call it wasted time. You know, I’ve wasted my youth, they have not learned the language, they have not done anything, and no wonder that there is the mental health crisis.”

#### 3.2.2 Social connections

In such an environment, it was considered difficult to develop social connections. Initially, asylum seekers were perceived to build ‘social bonds’ between people from similar backgrounds (e.g., shared culture, religion, or language). A community and integration manager said: “It depends on if they are form the same country, they speak the same language, it makes it easier to build a sense of community.” Another important element of bonding was connecting with people who shared similar experiences of violence and hardship, flight, and seeking asylum; people whom asylum seekers could trust, people who could understand them and, to some extent, feel their pain. An operations manager explained: “Mainly they think that people who are from the same sort of background or refugees or asylum seekers or their, for example, roommate or people who live in the community, they think that they are the ones that they can turn to get support and they think that they are the ones who can feel their pain, yeah?” Seeking “familiarity in a foreign land” was seen to create a sense of safety and security and a space to recover, particularly for those who struggled with mental health problems. The following quote makes this apparent: “You know creating a community among other migrants (…) really re-instills what they have lost. So yeah, I guess the sense of security really aids their recovery, especially migrants with mental health issues” (Community and integrations manager).

Less positively, such close connections were believed to limit asylum seekers’ and refugees’ social capital in that they isolated them from the wider British society. Several service providers characterized these bonds with words such as “clinging,” “sticking together,” “closed,” “inability to extend,” and “bundling up.” As such, bonds could turn into “barriers” as people held themselves back from going into and exploring the new environment around them. A mental health counselor said: “They are sort of clinging to their own culture. They want to be with their own people who speak the same language. (…) They are sort of afraid of the outside world, and any sort of acceptance of the British culture.” A participant with refugee background contemplated that people were, thus, partly excluding themselves from the community. Interestingly, service providers also recognized their own role in the creation of such closed social bonds in that most of their programs and activities were designed to bring people from similar backgrounds and with similar experiences together. Someone critically reflected that they might be culpable of contributing to a form of “ghettoization” among refugees.

Despite this self-critique, most viewed the role of organizations as one of ‘linkage’ between the “communities of similar experiences and interests” and the British host society as well as available services. Concretely, this involved activities that generated confidence and trust; supported with navigating the asylum, welfare, and health systems; helped with upskilling, including language learning; and provided financial and childcare support. A therapeutic services manager reflected: “So, support is very individual to each person, but I guess for them it’s just that recognition and understanding of what their needs are and I think that is something that we do (…) providing our holistic model. You know, if I get a client that is saying that ‘I am living in really terrible conditions and I need housing or warm clothes’, I will refer them to our advice team and our support team.”

Over time and with support, asylum seekers and refugees were perceived to widen their circles of interaction and built ‘bridges’ into host communities. The operations manager of an organization said: “At the beginning when they come, they are more focussed with their own group, refugees and asylum seekers as I say. But gradually, in the course of time, when they become more integrated, when they get lots of connection with non-refugees, then for them the definition of community will change.” In fact, most participants recognized that refugees and asylum seekers made great efforts to reach out to the wider community as they learned the language, made friends, volunteered, and started to study or to work. Becoming part of the wider community was considered crucial to rebuilding one’s life and support systems, but also an opportunity for “giving back” to those who had initially supported and hosted them. With time, people were seen to belong to multiple communities, something that was perceived to enhance their sense of belonging and overall wellbeing, including mental health. However, this was not understood to be a straightforward process. Building bridges was seen to be especially difficult for those who did not speak English considering that they could not easily partake in social life, education, and work. Moreover, host communities were, to some extent, described as unsupportive due to being “unfriendly,” “uncaring,” “hostile,” and “exclusionary,” and, thus, difficult to access. A community and integration manager said: “The United Kingdom has a lot of work to do in terms of attitudes and it’s that thing about social exclusion again, you can try and thrive but if you are excluded you can only get so far. So yeah, (…) there is hostility among the general public.” The person further clarified that hostility might not be shared by the majority of the population, but that those who harbored anti-immigration attitudes “[were] so loud that it seems like this is everyone’s opinion.” Feeling excluded and unwelcome was considered to be often hugely disappointing and hurtful to those who had arrived hopeful to restart their lives.

#### 3.2.3 Facilitators and markers of integration

Similar to the Home Office Indicators of Integration Framework, service providers mentioned work, housing, education, and health and social care as important for both integration and mental health facilitated mainly by language and communication, culture, safety and stability. In terms of employment, they emphasized that asylum seekers lacked the right to work while those with refugee status were often excluded from work due to prejudice and discrimination; poor English language skills; and/or not having their professional or educational certificates and diplomas recognized. This meant that many had to accept work that was either below their skill level or inadequate and poorly paid which was, in turn, linked to a poor quality of life, living in poverty, and feelings of disempowerment and depression. At the same time, several service providers highlighted that those who were employed worked incredibly hard, felt more empowered, had greater stability, considered themselves better integrated, and tried to give back to the communities that had helped them during their difficult time in the asylum process. Someone said: “Work is key as well when someone does find a job, they are much more empowered they are much more comfortable to be out there actually to see people and interact with people.”

Education was another important marker of integration. For younger people, education was perceived to be easier to access due to automatic school enrolment, and an opportunity to make friends with peers from various backgrounds. For adults and, especially women, accessing ESOL classes and further education was more challenging. A practitioner, working with Afghan women, reflected that the beneficiaries had to overcome strong fears and to learn how to build trust after a life of discrimination under the Taliban before being even able to attend English classes or other educational and psychosocial activities. Accessing further education such as college or university was explained to be difficult for most due to language barriers, difficulties navigating the online registration system, and the inability to finance studies or access competitive fellowships. A service provider said: “Funding for education is a massive barrier because I feel like that is a massive thing because if you are not able to go to college and learn a language then you are excluded” (Community and integration manager).

Accessing work and education was not only difficult because of the barriers mentioned above, but also because asylum seekers and refugees often struggled with their housing situation. Asylum seekers were frequently forced to move accommodation within cities or, as part of dispersal policies, between regions of the UK. Consequently, their social networks were fragmented and, in many cases, education and work interrupted. Accommodation itself was described as “inhumane,” “very poor,” “insecure” and “unsafe.” Some people were forced to live in flats or shared accommodation that was moldy, dysfunctional, and mouse and rat or insect infested. In some situations, women were perceived to be vulnerable due to unsafe housing conditions where they risked being harassed and assaulted by men. Some were scared to leave their rooms or apartments or felt uncomfortable using shared toilets, showers, and kitchens. An arts therapist recounted: “I once had an Eritrean girl say to me, ‘I was safer in Eritrea than I am in my house in Hammersmith somewhere.’ So, I mean there’s got to be a safe space for them to just put their head down at night and not to wake up, afraid and threatened.” Despite these mostly negative sentiments related to accommodation, a few practitioners mentioned that some refugees managed to find accommodation in safe neighborhoods with supportive and friendly neighbors who welcomed them, helped them navigate city life, and linked them to health and social care services.

Indeed, health and social care were perceived to be hugely important to help asylum seekers and refugees integrate into the community and experience good mental health. To be beneficial, it was recommended for health and social care be holistic and multi-layered so as to address the myriad basic needs people were facing including housing, language, welfare, education, work, social connection, and health support. However, only few organizations felt that they were capable to offer such holistic and multi-layered support. To mitigate this, some organizations had developed a well-functioning referral system that connected those in need with relevant health and social services. A therapeutic service manager illustrated this saying: “You know if I get a client that is saying that ‘I am living in really terrible conditions and I need housing or warm clothes’, I will refer them to our advice team and our support team who can help them with practical stuff and (…) access medical care [including] medication for basic things like diabetes, or blood pressure or thyroid.”

Less clear was how to provide effective mental health support due to numerous barriers and constraints. Most highlighted that asylum seekers and refugees faced difficulty accessing mental health and psychosocial support services without support to navigate the United Kingdom health system. The director of an organization explained: “Many people report issues with GPs because a GP will see you as you are assuming you are a British citizen and you understand the system and have the capacity to make the decision and have the social capital.” Other access barriers included shame and stigma, lack of trust, worry that confidentiality would not be maintained by service providers, and fear that disclosing a mental illness could lead to further social exclusion and even deportation. Those who managed to access mental health support were reported to struggle with expressing themselves in English which was not helped by the absence of translators and cultural mediators. A policy and practice advisor explained: “There’s a real lack of (…) cross-cultural understanding of how people perceive mental health and wellbeing in other cultures and the language they use to talk about it and how they would present with issues as well.”

### 3.3 Thriving in the context of hostility

All service providers explained that for community integration to be meaningful, it had to go beyond mere “survival.” It had to empower asylum seekers and refugees to “thrive.” Thriving was considered to manifest in asylum seekers and refugees fulfilling their aspirations and dreams, having agency, reaching their potential, and leading the lives they valued. A communications officer and arts director stated, for example: “(…) thriving is the moment when you start to take control. When you have agency, when you have an idea, a goal, an image for my future, and I know at least the first few steps toward it. And I think that to me is thriving.”

Thriving was described as a multi-layered concept. On the individual level it was located within the person as a kind of inner strength or “resource” that helped them to “bounce back,” “have hope,” and “be determined to start a new life.” This strength or resource was one that could be mobilized by people to meet “external opportunities” and “impact on the world around them.” An organization’s director explained: “it is knowing that you have the internal resources and confidence and that they meet external opportunities and there’s that sort of magic moment where it’s like wow I have done something that has impact.” Thriving was seen to have a course of direction as it propelled people forward despite “obstacles,” “setbacks,” and “struggles.” For people to move forward against the odds required an “I can do it” attitude and “not being in limbo” that is, stability, knowing that one had the right to stay in the UK, and being given opportunities.

Yet, thriving was not merely seen as innate to the individual. It was recognized that no one could thrive on their own. Rather, it was a reciprocal process whereby people supported each other within an environment conducive to flourishing. The director of an organization said:

And then being given opportunities to achieve your aspirations and to have people to support you along the way. And to be able to support others as well through that process. I think it's also about the environment that you're in, whether you know to be able to live in a place where it's not a squalid or a tiny room and you are looked after and have access to parks and greenery you know, like in the countryside, and you have the opportunity to travel and move around.

Support connected to thriving was thus not only social, but also material in that it was considered impossible to thrive without financial support, educational and work opportunities, appropriate living conditions and childcare, and healthcare in place.

Thriving, it becomes apparent, was linked to, but went beyond integration, and practitioners saw a role for themselves in helping asylum seekers and refugees in their journey. Considering this, practitioners had a very realistic view on thriving explaining that they perceived it as extremely difficult in an environment where policies and political attitudes were anti-immigration and where rights were extremely limited especially for those in the asylum system. A policy and practice advisor pointedly said: “It’s so challenging and with the hostile environment and with the way the Home Office immigration and asylum process works, it’s very, very difficult and very challenging to be able to thrive.” Similarly, a therapist said when asked what would help asylum seekers and refugees to thrive: “A fairer immigration system. A fairer, more efficient immigration system would work. Stopping the hostile environment policy which has been kind of implemented across the board.”

## 4 Discussion

The study results show that the current asylum system severely undermines efforts to support asylum seekers and refugees with their integration. All participants highlighted that asylum seekers and refugees lacked experienced poor quality of life and faced structural challenges to build meaningful social connections; to have access education, fair employment and good work; to achieve good mental health and wellbeing; and to be able to thrive. The domains of the United Kingdom Home Office’s Indicators for Integration Framework are, thus, far from being met. Indeed, service providers highlighted that asylum seekers and refugees lack basic rights and entitlements; do not experience safety and security that would allow them to make a place for themselves in the society due to the long backlog of asylum applications, unstable living conditions, and destitution; and receive only very limited support in their integration whether it is through language training, education and work opportunities, housing, or leisure.

The little community integration support that is available, is mainly provided by local communities and third sector organizations. Service providers highlighted their role in (a) providing health and mental health care, legal support and translation services, language training, and material support to cover basic needs such as food, clothing, and accommodation; (b) helping newcomers to navigate the welfare and health systems and link asylum seekers and refugees with various other support services; and (c) giving asylum seekers and refugees a sense of community by bringing people with similar backgrounds and experiences together in a trusted environment. At the same time, they also mentioned limitations to their work mainly related to the fact that they are grossly underfunded and, in many cases, understaffed. Self-reflected, some participants also mentioned that their integration support mainly rested on establishing ‘social bonds’ rather than fostering ‘social bridges’ into the wider community which would allow asylum seekers and refugees to benefit from potential social capital and upward social mobility. While this was recognized as a limitation, study participants also acknowledged that the community itself was not always receptive to newcomers exhibiting exclusionary attitudes such as racism and xenophobia which, in and of themselves, are social determinants linked to poor health, including poor mental health.

**Figure 2:**
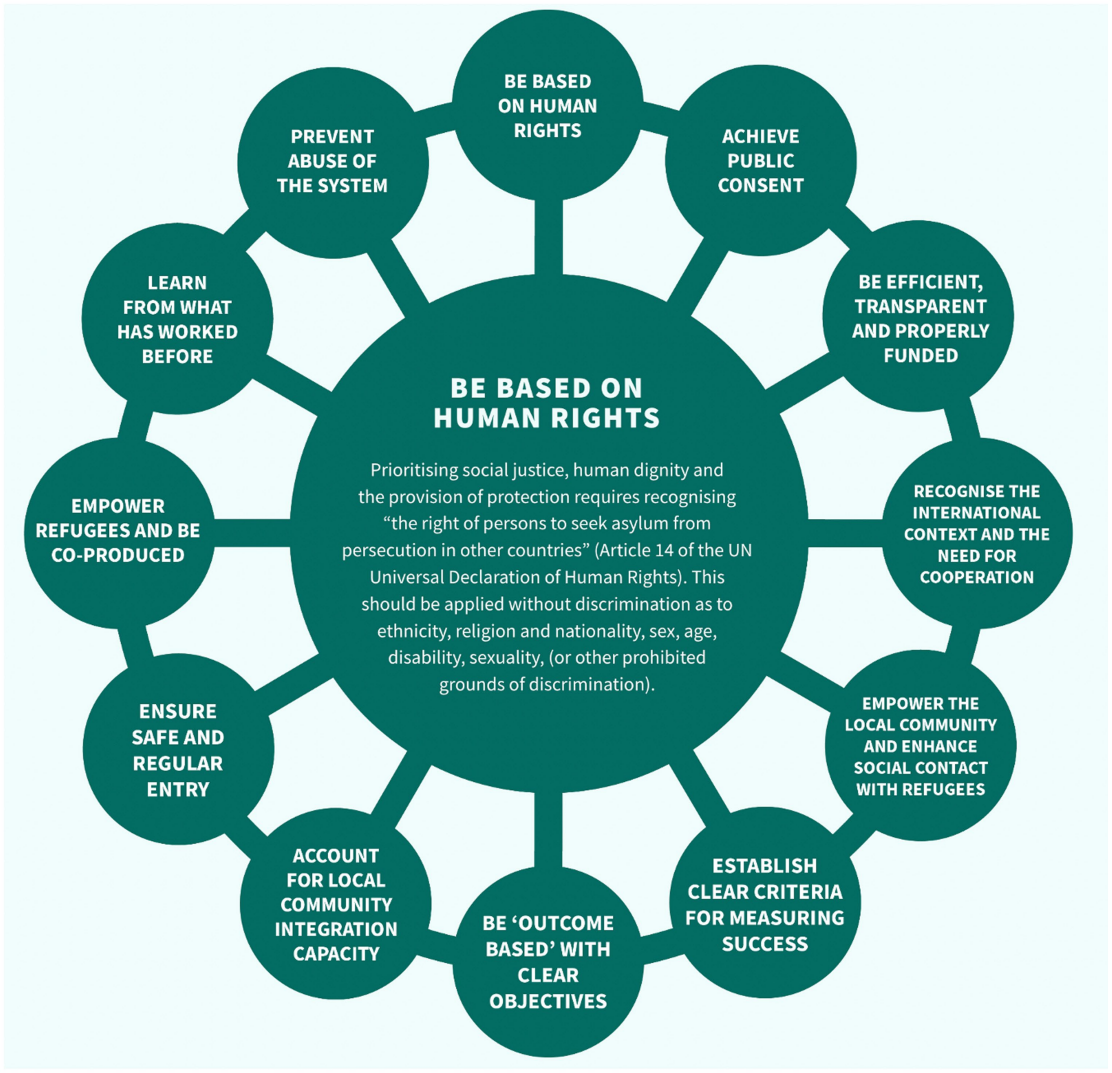
Key components for an alternative asylum system proposed by the Commission of the Integration of Refugees (the figure is interactive and can be accessed here: https://refugeeintegrationuk.com/our-mission/).

To support asylum seekers and refugees with community integration, achieving a good quality of life, being able to thrive, and having improved mental health will require to address structural inequalities that are tightly woven into our social fabric. First, this will require a thorough reform of the current asylum system based on human rights, social justice, human dignity, and the provision of protection with full recognition of the “the right of persons to seek asylum from persecution in other countries” (Article 14 of the UN Universal Declaration of Human Rights). It is worth highlighting the work of the ongoing Commission on the Integration of Refugees which proses an alternative to the current asylum system summarized in the Figure 2 (29).

Second, to provide meaningful integration support, government and third-sector organizations should implement and carry out needs assessments among asylum seekers and refugees focussing on key social determinants that ensure giving children the best start in life; enabling young people to maximize their capabilities and control their lives; creating fair employment and good work; ensuring a healthy standard of living; creating and developing healthy and sustainable places and communities; and strengthening the role and impact of ill health prevention (30). As part of this endeavor, the Department of Health’s commissioned Joint Strategic Needs Assessment (JSNA) toolkit could be adapted and validated. The JSNA proposes a process by which local authorities and Clinical Commissioning Groups assess the current and future health, care, and wellbeing needs of the local community to inform local decision making (31). Local decision making, in turn, is to be based on evidence including key population-level data as well as social and place-based determinants (e.g., housing quality, environment, employment, education attainment, benefit uptake, vulnerable groups, crime and disorder, and community cohesion); lifestyle determinants (e.g., exercise, smoking, diet, alcohol, drug use); epidemiology (e.g., morbidity, mortality, life expectancy, long-term conditions, disease prevalence, immunization uptake rates); service access and utilization and evidence of effectiveness; and community perspectives. By adapting and validating the toolkit for asylum seekers and refugees, needs could be assessed and addressed so as to improve their quality of life and health and mental health outcomes strategically and meaningfully.

Third, asylum seekers and refugees should benefit directly from the NHS Inclusion Health framework which is directed at people who are socially excluded and typically experience multiple interacting risk factors for poor health, such as stigma, discrimination, poverty, violence, and complex trauma (32). People in ‘inclusion health groups’ explicitly include “vulnerable migrants and refugees” with the recognition that they tend to have poor experiences with healthcare services which leads them to avoid contact with the NHS despite having high needs. The framework itself focuses on five principles for action to ensure those typically excluded can experience better health outcomes: (1) commit to action on inclusion health; (2) understand the characteristics and needs of people in inclusion health groups; (3) develop the workforce for inclusion health; (4) deliver integrated and accessible services for inclusion health; and (5) demonstrate impact and improvement through action on inclusion health. To achieve this, it is recommended to work in partnership with affected people and communities and in close collaboration with other government departments and agencies.

Fourth, to address the systemic racism and xenophobia experienced by asylum seekers and refugees in the healthcare system, it is important to acknowledge that marginalized communities have been, and continue to be, subjected to harm and unjust treatment by our institutions (33). Thus, anti-racist practice needs to become explicitly part of institutional culture. NHS England has taken an important step into this direction by adopting the mandatory “Patient and carer race equality framework” (PCREF). The framework stipulates that it will “support trusts and providers on their journeys to becoming actively anti-racist organizations by ensuring that they are responsible for co-producing and implementing concrete actions to reduce racial inequalities within their services” (34). Improvements are predicted to occur in three key domains including leadership and governance; data generation; and feedback mechanism. Psychiatric practice, more specifically, could be specifically guided by the antiracism principles and recommendations outlined by Fani et al. (33) including (1) introspective practices that enhance knowledge of privilege and latent bias among clinicians; (2) cultural considerations in assessment, diagnosis, and treatment planning; and (3) addressing barriers to mental health care.

In order for any of these recommendations to have their intended positive effects on integration, quality of life, thriving, and mental health among asylum seekers and refugees, they need to be grounded in a participatory approach. That is, assessments of needs, development of meaningful and diverse support mechanisms, and monitoring and evaluation of such supports must involve various stakeholders, including asylum seekers and refugees, communities hosting them, employers and educators, housing associations, social workers, health providers, and policy makers among others (35). Inspired by principles of participatory action research, such inclusive working should be based on (1) social change to enable action that leads to systemic, social, and behavioral changes; (2) participation that ensures that the agenda is driven by those who have a stake in the issue; (3) power of knowledge whereby knowledge is carefully and purposefully produced with diverse stakeholders; and (4) collaboration by expanding the emphasis from action and change to collaborative policy making and service provision starting with program planning and including implementation and evaluation (36). The Indicators of Integration Framework could play an important role in this by further evidencing meaningful asylum seeker and refugee integration and its effects on people’s quality of life, thriving, and health outcomes in the long-term.

### 4.1 Limitations

This study has several key limitations. Although appropriate for a qualitative study, the sample is relatively small and focuses mainly on experiences of service providers working in charities based in London. It needs to be recognized that needs and support provision differ across the United Kingdom and that the devolved governments of Scotland, Wales, and Northern Ireland have different approaches to asylum seeker and refugee integration, social support, and healthcare. Importantly, this research reflects views of those providing support rather than asylum seekers and refugees themselves. In the spirit of participatory working, it will be important to carry out similar research in collaboration with asylum seekers and refugees to explore their lived experience, needs, and demands for meaningful social and (mental) health support.

## Data availability statement

The datasets presented in this article are not readily available because data will not be available as it will be impossible to guarantee anonymity.

## Ethics statement

The study involving humans was approved by King’s College London Research Ethics Committee (reference number: MRA-19/20–20750). The study was conducted in accordance with the local legislation and institutional requirements. The participants provided their written informed consent to participate in this study. Written informed consent was obtained from the individual(s) for the publication of any potentially identifiable images or data included in this article.

## Author contributions

HK: Conceptualization, Formal analysis, Funding acquisition, Investigation, Methodology, Project administration, Software, Writing – original draft, Writing – review & editing.

## Footnotes

^1^The United Kingdom distinguishes between refugees and asylum seekers with different rights and obligations connected to each group. Refugees are persons who have their asylum claim accepted and are the defined based on the 1951 UN Refugee Convention as a person who is unable or unwilling to return to their country of origin “owing to a well-founded fear of being persecuted for reasons of race, religion, nationality, membership of a particular social group, or political opinion.” Asylum seekers are persons who are seeking international protection and have applied for refugee status under the convention, but whose claims have not yet been determined (5).

^2^ https://www.kcl.ac.uk/research/mhpss-directory
